# Type 2 IgG Memory B Cells as Precursors of IgE Plasma Cells

**DOI:** 10.1111/all.16473

**Published:** 2025-01-11

**Authors:** Larissa Nogueira de Almeida, Janina Petry, Christopher C. Udoye

**Affiliations:** ^1^ Institute of Nutritional Medicine University of Lübeck and University Hospital Schleswig‐Holstein Lübeck Germany; ^2^ Institute for Systemic Inflammation Research University of Lübeck and University Hospital Schleswig‐Holstein Lübeck Germany

Immunoglobulin E (IgE) is a major mediator of allergic reactions and can activate mast cells and basophils via the high‐affinity IgE receptor. Some IgE‐mediated allergies can last a lifetime, but IgE‐secreting plasma cells (PCs) and IgE+ memory B cells (MBCs) are rare, leading to questions about the origin of allergen‐specific IgE antibodies (Abs). In recent years, studies have suggested that allergen‐specific B cell memory is predominantly held by IgG(1) MBCs, which can become IgE‐secreting PCs during a recall response [1, 2]. A recent article by Koenig and colleagues proposed a novel class‐switched MBC population with type 2 immune markers, which they termed MBC2, as a major progenitor of IgE PCs [3]. The most important findings of their work are highlighted in Figure 1.

**FIGURE 1:**
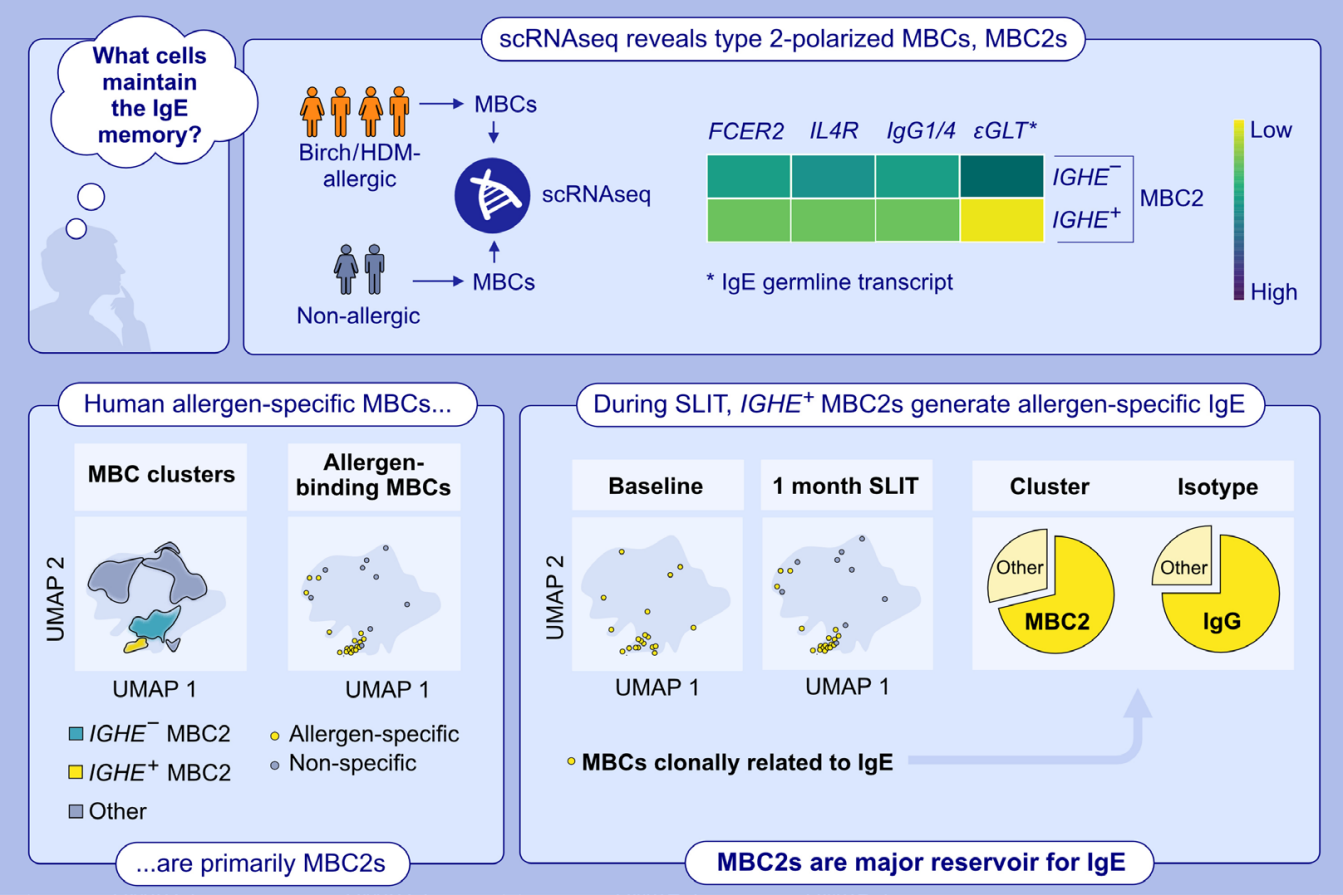
Upper panel, *IGHE+* MBC2s express type 2 immune markers. scRNA‐seq was performed using MBCs selected from allergic and non‐allergic individuals. Lower left panel, Allergen‐specific MBCs are primarily MBC2s. When plotted onto the MBC atlas UMAP, most allergen‐specific transcriptomes (from allergen tetramer binding MBCs) clustered with MBC2 cells. Lower right panel, Allergen‐specific MBC2s are precursors of SLIT‐induced IgE+ PC clones. Clonal relationship between IgE+ plasma cells and MBC2 at baseline and after 1 month of SLIT. Most of IgE‐related MBCs isotypes identified in antibody‐encoding reads were IgG. MBC, memory B cell; PC, plasma cell; scRNA‐seq, single‐cell RNA sequencing; SLIT, sublingual immunotherapy; UMAP, Uniform Manifold Approximation and Projection.

Using single‐cell RNA sequencing (scRNA‐seq), the authors identified 21 MBC clusters in both allergic and non‐allergic subjects (Figure 1, upper panel). Two MBC clusters, collectively termed MBC2, expressed high levels of the low‐affinity IgE receptor (CD23) and receptors for the type 2‐associated cytokines IL‐4 (IL4R) and IL‐13 (IL13RA1). MBC2s contained two subsets, *IGHE*+ and *IGHE*‐ MBC2s, which differed in the expression of IgE germline transcripts (εGLTs), but barely expressed productively rearranged IgE transcripts. Instead, both subsets expressed predominantly IgG1 B cell receptors (BCRs), and a relatively high proportion of IgG4 in the *IGHE*+ MBC2s. Importantly, the frequency of *IGHE+* MBC2 cells was independent of the allergic status and did not correlate with allergic disease. The authors confirmed this MBC2 phenotype at the protein level with additional elevated expression of CD40 and HLA‐DR/DQ genes and a unique down‐regulation of the inhibitory IgG receptor FcγRIIB compared to other MBC subsets.

The authors also found a similar IL‐4‐dependent CD23hi/IL‐4Rαhi MBC2‐like population in mice, which emerged in the context of type 2 but not type 1 sensitization, with 2% of antigen‐specific, class‐switched MBCs showing the *IGHE+* MBC2 phenotype (the majority of which expressed an IgG1 BCR) upon type 2 sensitization. Importantly, the authors also showed that antigen‐specific MBC2 generation in mice was primarily dependent on germinal center (GC) reactions, with less than 10% of antigen‐specific MBC2s appearing to develop independently of GCs.

Finally, an analysis of birch allergic patients undergoing sublingual immunotherapy (SLIT) revealed a clonal connection between IgE‐producing PCs analyzed 1 month after the start of SLIT and MBC2s analyzed before or 1 month after the start of SLIT, although it remained unclear what percentage of IgE+ PC clones were found in MBC2 clones (Figure 1, lower panel). Together with an independent article in the same issue [4], Koenig and colleagues provide insights into allergen‐specific B cell memory by identifying class‐switched *IGHE*+ MBC2s with a strong type 2 phenotype, which appears to be generated mainly in GCs, as potential precursors of IgE PCs in allergen‐specific recall responses in allergy, but also after allergen‐specific immunotherapy (AIT). Their work characterizes in detail the phenotype of such MBC2s for the first time and raises multiple questions that should be explored in the near future.

Notably, it remains unclear which factors influence *IGHE*+ MBC2s to switch to IgE‐ instead of IgG1‐ or IgG4‐secreting PCs during an allergic or AIT recall response, especially considering that *IGHE+* MBC2s may also be present in non‐allergic individuals. Furthermore, it remains unclear what proportion of IgE‐secreting PCs develop directly from re‐activated *IGHE+* MBC2s or instead from IgE+ MBCs (expressing a functional IgE BCR), or whether all or some of the re‐activated *IGHE+* MBC2s or instead IgE+ MBCs re‐enter the GC to become “long‐lived” IgE‐secreting PCs, or what proportion of IgE‐secreting PCs develop independently of GCs and GC‐derived MBCs. Accordingly, IgE+ GC B cells have been described as precursors of IgE+ MBCs and IgE‐secreting PCs [5], and long‐lived IgE‐secreting PCs have also been reported [6].

Interestingly, it has been suggested in the context of IgG Abs that direct re‐activation of (IgG+) MBCs induces IgG+ PCs that, like extrafollicular IgG+ PC responses, generate short‐lived and highly galactosylated and sialylated IgG Abs, whereas re‐activated MBCs re‐entering the GC generate IgG+ PCs that produce long‐lived and less galactosylated and sialylated IgG Abs, with the level of galactosylation and sialylation of the latter IgG Abs further dependent on co‐stimulatory signals in the GC reaction, and that these different IgG Abs may play different roles [7, 8]. Whether these differences also play a role for IgE is still unclear, but individual IgE compositions of such different pathways may influence the overall quality and effector function of IgE.

The identification of class‐switched allergen‐specific *IGHE+* MBC2s supports and concretizes recent suggestions that at least part of IgE‐secreting PCs are derived from (IgG+) MBCs. (*IGHE+*) MBC2s may therefore represent a potential biomarker and therapeutic target for IgE‐mediated allergic diseases, which requires further investigation.

## Conflicts of Interest

The authors declare no conflicts of interest.

## Data Availability

Data sharing is not applicable to this article as no new data were created or analyzed in this study.
